# Supplementary material for: Sex-Specific Effects on Total Body Fat Gain with 4-Week Daily Dosing of Raspberry Ketone [4-(4-Hydroxyphenyl)-2-butanone] and Ketogenic Diet in Mice
Source: Nutrients. 2023 Mar 28;15(7):1630. doi: 10.3390/nu15071630 (PMC10097242; doi:10.3390/nu15071630)
Supplement: Supplementary file 1 [file nutrients-15-01630-s001.zip › nutrients-2239883-supplementary.pdf]

nutrients

MDPI

Article

Sex-Specific Effects on Total Body Fat Gain with 4-Week Daily Dosing of Raspberry Ketone [4-(4-Hydroxyphenyl)-2-butanone] and Ketogenic Diet in Mice

Lihong Hao and Nicholas T. Bello *

Department of Animal Sciences, School of Environmental and Biological Sciences, Rutgers, The State University of New Jersey, New Brunswick, NJ 08901, USA; haoli@sebs.rutgers.edu
* Correspondence: ntbello@sebs.rutgers.edu

Citation: Hao, L.; Bello, N.T. Sex-Specific Effects on Total Body Fat Gain with 4-Week Daily Dosing of Raspberry Ketone [4-(4-Hydroxyphenyl)-2-butanone] and Ketogenic Diet in Mice. *Nutrients* **2023**, *15*, 1630. https://doi.org/10.3390/nu15071630

Academic Editor: Fabio Galvano

Received: 8 February 2023
Revised: 16 March 2023
Accepted: 21 March 2023
Published: 28 March 2023

Copyright: © 2023 by the authors. Licensee MDPI, Basel, Switzerland. This article is an open access article distributed under the terms and conditions of the Creative Commons Attribution (CC BY) license (https://creativecommons.org/licenses/by/4.0/).

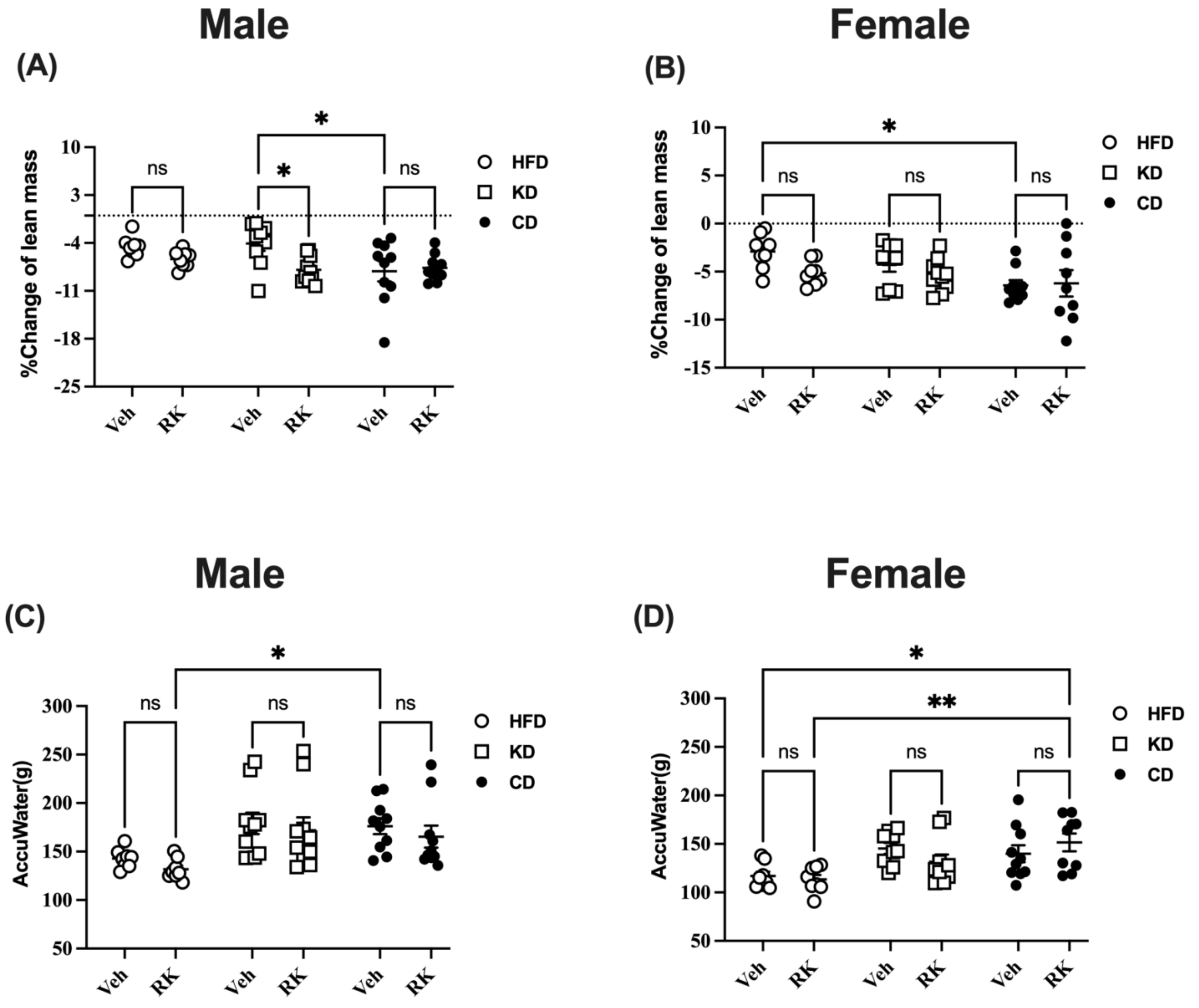

Figure S1. Percentage change of lean mass (A, B) and accumulated water intake (C, D) in overweight/obese male and female mice after 4 weeks of daily dosing and diet feeding. * $p < 0.05$; ** $p < 0.01$; ns, not significant. Two-way analysis of variance (ANOVA) followed by Newman Keul post hoc tests when justified; $p < 0.05$ was considered statistically significant. N=8-10 mice/group. high fat diet (45%fat), HFD; ketogenic diet (80%fat), KD; control diet (10%fat), CD; vehicle, Veh; raspberry ketone, RK; accumulated water intake, AccuWater.

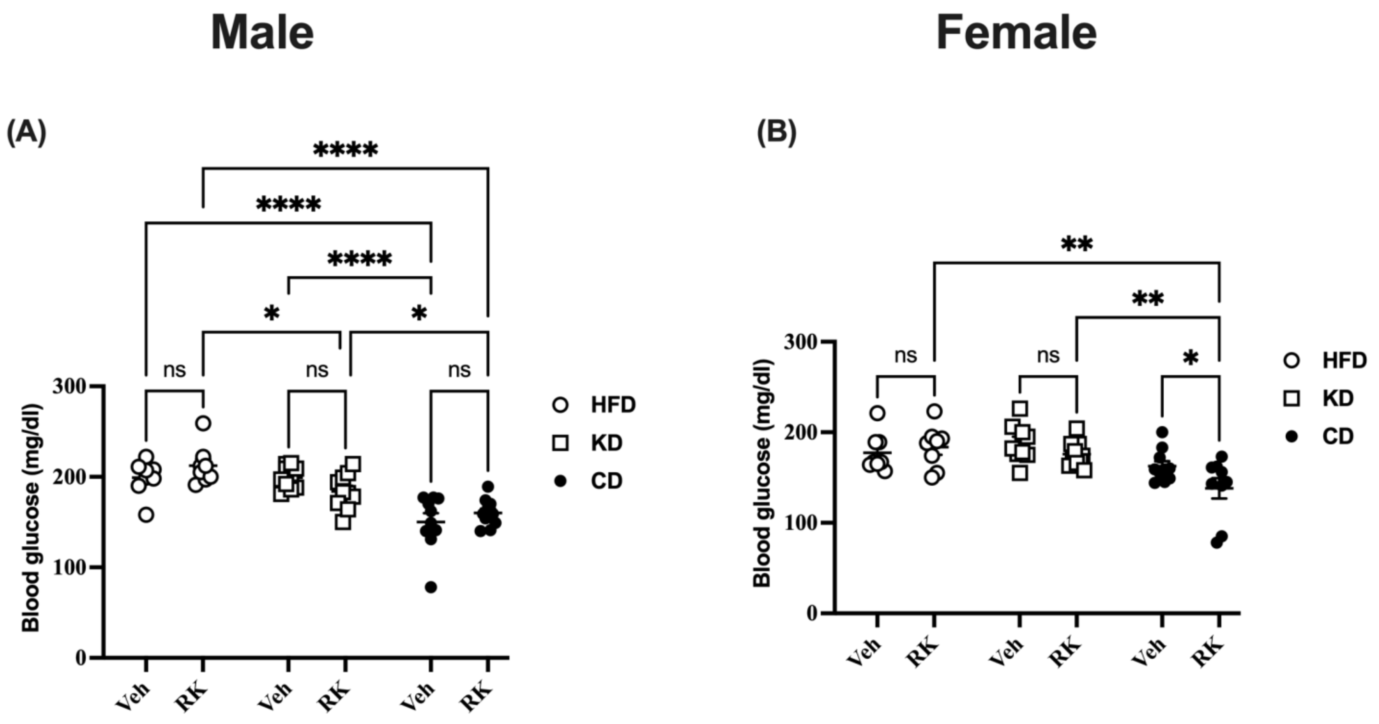

Figure S2. Baseline blood glucose levels in overweight/obese male (A) and female (B) mice after 3 weeks of daily dosing and diet feeding. * $p < 0.05$; ** $p < 0.01$; **** $p < 0.0001$; ns, not significant. Two-way analysis of variance (ANOVA) followed by Newman Keul post hoc tests when justified; $p < 0.05$ was considered statistically significant. N=8-10 mice/group. high fat diet (45%fat), HFD; ketogenic diet (80%fat), KD; control diet (10%fat), CD; vehicle, Veh; raspberry ketone, RK.

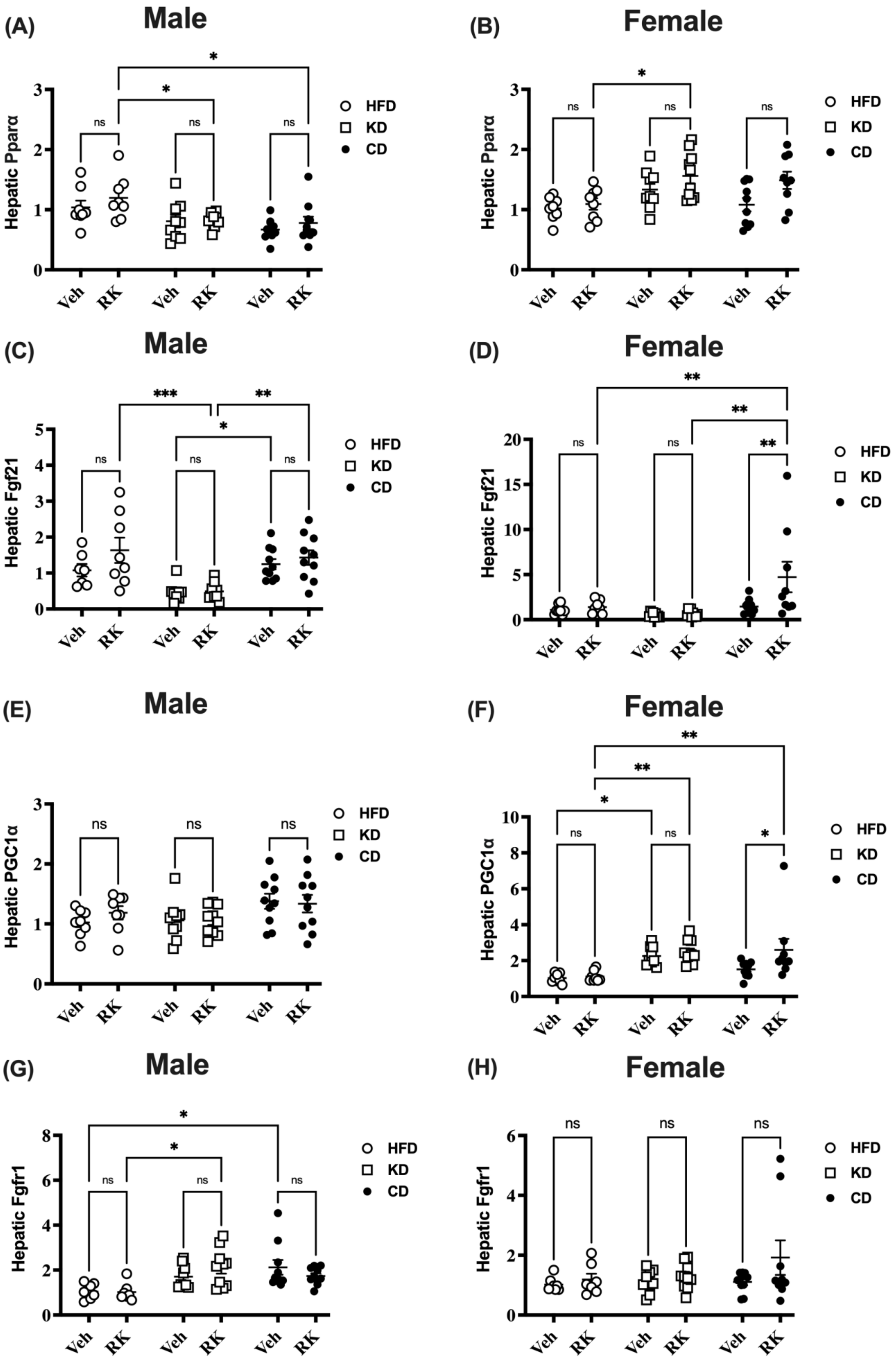
(A)
Male
Hepatic Pparα
HFD
KD
CD
Veh RK Veh RK Veh RK
ns
*
(B)
Female
Hepatic Pparα
(C)
Male
Hepatic Fgf21
***
**
*
(D)
Female
Hepatic Fgf21
(E)
Male
Hepatic PGC1α
(F)
Female
Hepatic PGC1α
(G)
Male
Hepatic Fgfr1
(H)
Female
Hepatic Fgfr1

Figure S3. Hepatic gene expression of peroxisome proliferator-activated receptor alpha (*Pparα*) (A, B), fibroblast growth factor 21 (*Fgf21*) (C, D), peroxisome proliferator-activated receptor-gamma coactivator (PGC)-1alpha (*PGC1α*) (E, F) and fibroblast growth factor receptor (*Fgfr1*) (G, H) in overweight/obese male and female mice after 4 weeks of daily dosing and diet feeding. * $p < 0.05$; ** $p < 0.01$; *** $p < 0.001$; ns, not significant. Two-way analysis of variance (ANOVA) followed by Newman Keul post hoc tests when justified; $p < 0.05$ was considered statistically significant. N=8-10 mice/group. high fat diet (45%fat), HFD; ketogenic diet (80%fat), KD; control diet (10%fat), CD; vehicle, Veh; raspberry ketone, RK.
